# *Staphylococcus epidermidis* induced toxic shock syndrome (TSS) secondary to influenza infection

**DOI:** 10.1186/s12879-023-08487-3

**Published:** 2023-09-06

**Authors:** Charis Armeftis, Andreas Ioannou, Theodorakis Lazarou, Achilleas Giannopoulos, Efrosyni Dimitriadou, Kostantinos Makrides, Zoi Dorothea Pana

**Affiliations:** 1 Ygia Polyclinic Private Hospital, 21 Nafpliou Str, Limassol, 3025 Cyprus; 2 Medical School, European University, 6, Diogenous Str, Engomi, 2404 Nicosia Cyprus; 3 Infection Control and Antimicrobial Stewardship Medical School, EUC, Engomi, Nicosia Cyprus

**Keywords:** Case report, Staphylococcus epidermidis, Toxic shock syndrome, Influenza, Superinfection

## Abstract

**Background:**

To date, few cases of TSS caused by coagulase negative (CoN) *staphylococci* have been reported in the literature. Recent data show that CoN *staphylococci* are capable of secreting a number of enterotoxins and cytotoxins, normally produced by *S. aureus*. Herewith, we describe a case of TSS caused by *Staphylococcus epidermidis* with a favorable outcome.

**Case presentation:**

We report a case of a 46-year-old man who developed TSS from *S. epidermidis*. The patient was admitted for a 7-day history of general malaise and headache following a recent influenza infection and a 3-day history of vomiting, diarrhea, diffuse erythroderma, and fever. The main laboratory findings on admission were leukopenia (WBC 800/mm3), thrombocytopenia (Plt count 78.000/mm3), elevated urea, creatine levels and increased inflammatory markers (CRP 368 mg/ml). The patient had clinical and radiological evidence of pneumonia with chest computed tomography (CT) showing diffuse bilateral airspace opacifications with air bronchogram. On the second day, a methicillin resistant *S. epidermidis* (MRSE) strain was detected in both sets of blood cultures, but the organism was unavailable for toxin testing. All other cultures and diagnostic PCR tests were negative. His clinical signs and symptoms fulfilled at that stage four out of five clinical criteria of TSS with a fever of 39 °C, diffuse erythroderma, multisystem involvement and hypotension. On the same day the patient was admitted to the ICU due to acute respiratory failure. The initial treatment was meropenem, vancomycin, levofloxacin, clindamycin, IVIG and steroids. Finger desquamation appeared on the 9th day of hospitalization, fulfilling all five clinical criteria for TSS.

**Conclusions:**

To our knowledge, this is the first adult case with TSS induced by CoNS (MRSE) secondary to an influenza type B infection, who had favorable progression and outcome. Further research is warranted to determine how TSS is induced by the CoNS infections.

## Background

A common cause of severe influenza pathogenesis is superinfection with bacterial pathogens, most frequently, *Staphylococcus aureus* and *Streptococcus* pneumoniae [1]. Regardless of the infectious agent, bacterial superinfections are associated with increased morbidity and mortality rates during influenza pandemic and epidemic outbreaks [2]. According to the literature, the resolution of inflammation following an episode of influenza infection, is regarded as a period of enhanced susceptibility to several respiratory bacterial infections, resulting in bacterial superinfection, bacterial pneumonia and bacterial dissemination from the lungs [3]. This co-pathogenesis is characterized by complex interactions between co-infecting pathogens and the host, leading to dysregulation of immune responses and delays in a return to homeostasis [4].

Coagulase-negative *staphylococci* are under-appreciated as a cause of severe clinical conditions, including TSS [5–7]. Previous studies have shown that coagulase-negative staphylococci do not produce the toxin TSST-1, but they are capable of secreting a number of staphylococcal enterotoxins and cytotoxins, normally produced by *S. aureus* [6, 7]. Recently Staphylococcal enterotoxins A, D, and E were detected in *Staphylococcal epidermidis* strains playing the role of superantigens [11]. An immune reaction to the proliferation of CoNS organisms that causes cytokine activation has been proposed as an additional possible pathophysiological mechanism of CoNS induced TTS [8]. In particular, strains of *Staphylococcus epidermidis* isolated from patients with toxic shock symptoms have been reported to carry genes related to stimulation of human monocytes fostering the production of the cytokines TNF alpha, IL-1 beta and IL-6 [8]. To date, the incidence of TSS induced by CoNS is largely not known and only few cases have been reported in the literature [8–11].

### Case presentation

**Fig. 1:**
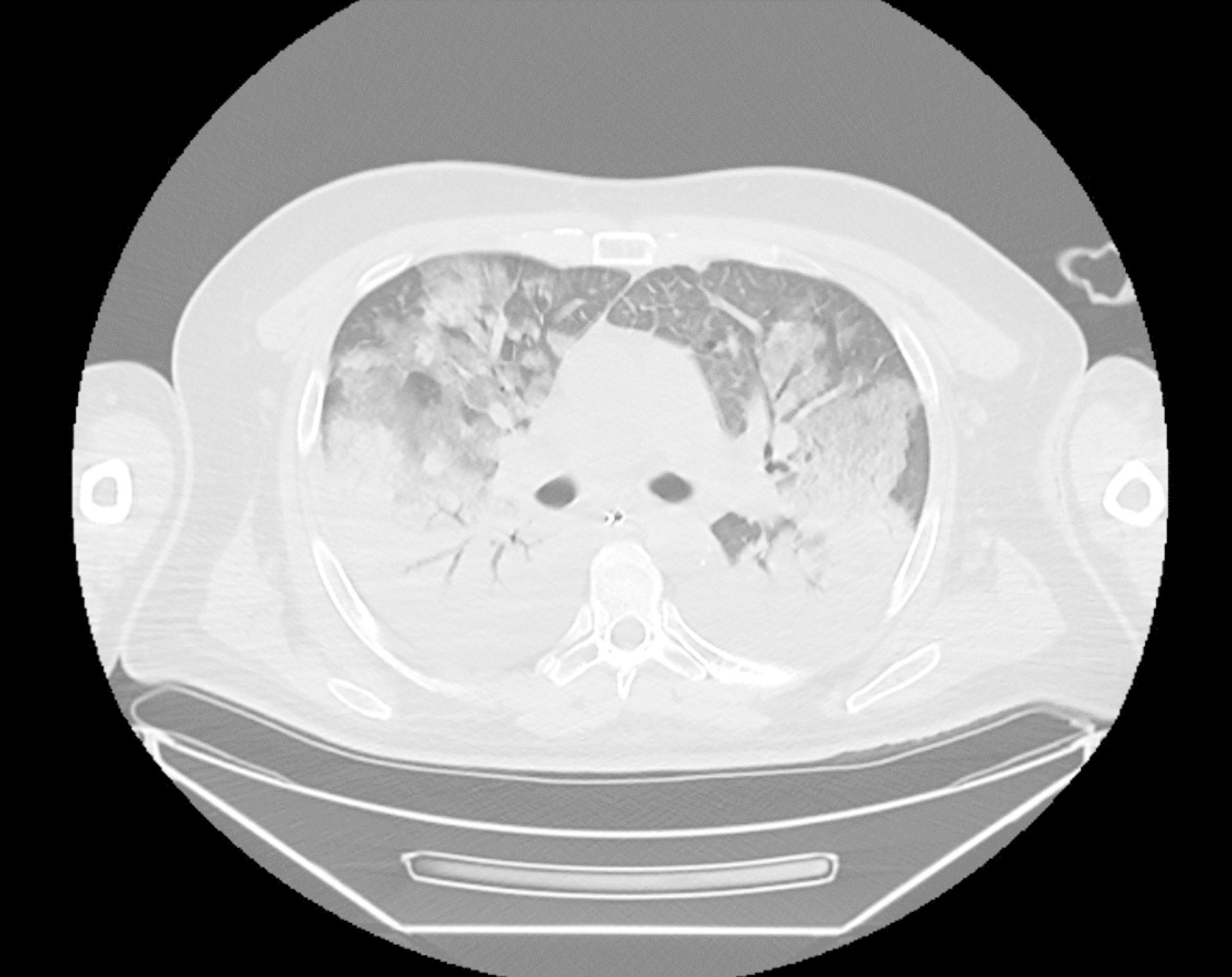
Chest computed tomography on admission

We report a case of a 46-year-old man, without chronic underlying conditions, who developed TSS from *S. epidermidis* following an influenza type B infection. The patient was brought to the hospital on an ambulance. He was admitted to the hospital for a 7-day history of general malaise, headache following a recent influenza type B infection, who gradually developed vomiting, diarrhea, diffuse erythroderma and fever during the last three days before admission. The consciousness level on admission, body temperature, blood pressure, pulse rate, respiratory rate, and peripheral oxygen saturation were E3V4M6, 39 °C, 87/50 mmHg, 130 beats/minute, 38 breaths/minute, and 91%, respectively. The main laboratory findings on admission were as following: leukopenia (WBC 800/mm3), thrombocytopenia (Plt count 78,000/mm3), elevated urea, creatine levels and increased inflammatory markers (CRP 248 mg/ml) [Table 1]. The patient had clinical and radiological evidence of pneumonia with chest computed tomography (CT) showing diffuse bilateral airspace opacification [Fig. 1]. Head CT was normal.

**Fig. 2:**
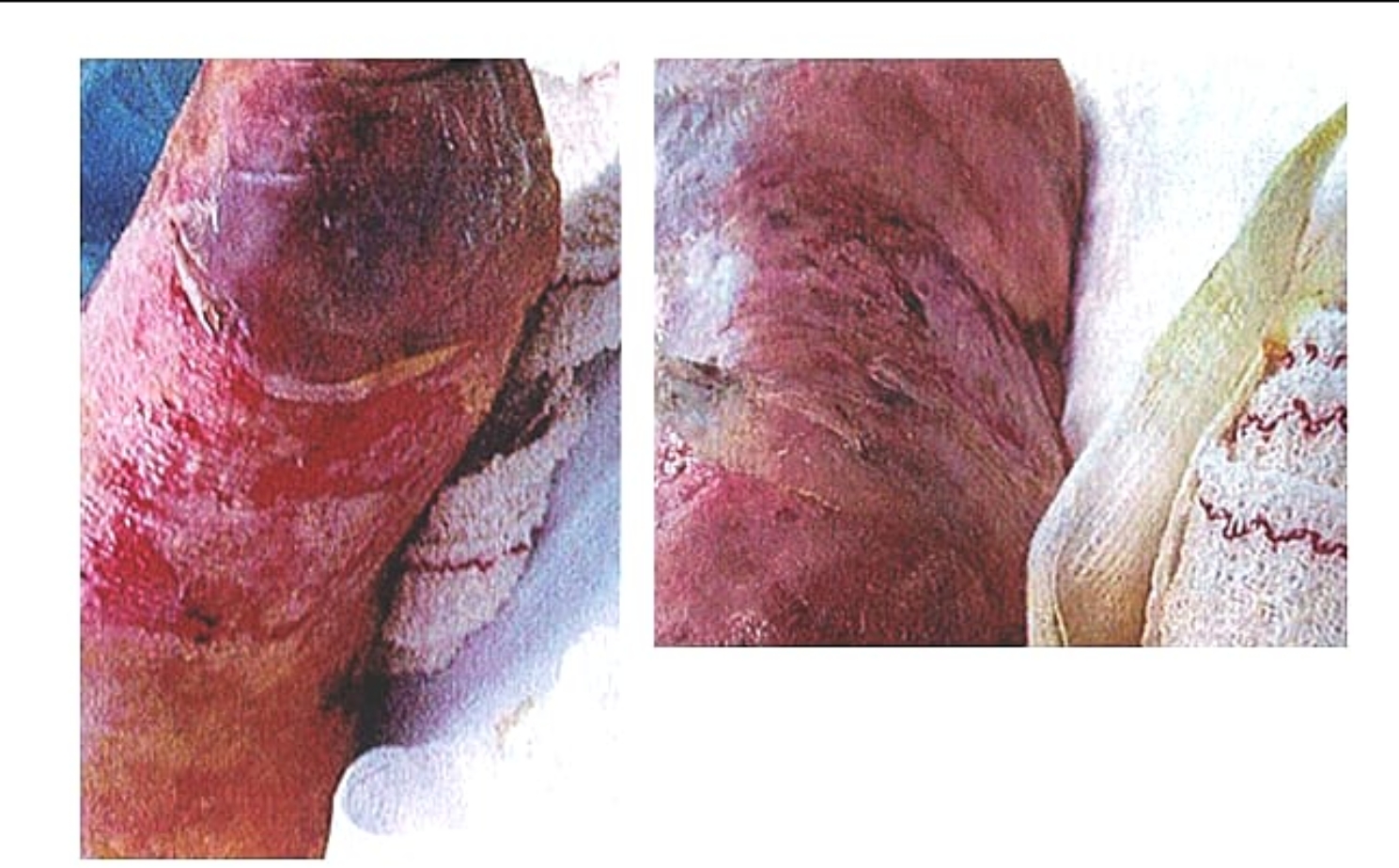
Foot desquamation on 9th day of hospitalization

On the second day, both sets of blood cultures obtained from two different peripheral venous sites (left and right arm) were positive for *S. epidermidis*, but the organism was unavailable for toxin testing. According to the antibiogram, the strain was resistant to methicillin (Methicillin resistant *Staphylococcus epidermidis*, MRSE). Urine cultures were negative. After intubation for sedation and procedures, both sputum and endotracheal cultures showed growth of only normal upper respiratory flora. The results of the full respiratory pathogen PCR panel, including SARS-CoV-2, were negative. The patient’s condition deteriorated on the same day, presenting acute respiratory failure, and he was admitted to the ICU. His clinical signs and symptoms fulfilled at that stage four out of five clinical criteria of TSS with a fever of 39 °C, diffuse erythroderma, multisystem involvement and hypotension [Table 2] [12]. Based on the clinical diagnosis of probable CoNS induced TTS and the critical condition of the patient, the following treatment was initiated during the first days of hospitalization: meropenem (1 g every 8 h), vancomycin (1 g every 12 h), levofloxacin (750 mg 24 h), clindamycin (600 mg every 8 h). The choice of the initial empirical antibiotic therapy was based on local guidelines due to high antimicrobial resistant rates (AMR) rates. The addition of clindamycin was based on the high suspicion of TTS. IVIG (2 g/kg) and hydrocortisone (100 mg every 8 h) were administrated to the patient due to high suspicion of TTS The mechanism responsible for the efficacy of gamma-globulin (IVIG) therapy may be neutralization of the circulating toxins, inhabitation of TNF-alpha production via nonspecific inhabitation of monocyte or T-cell activation, or inhibition of other staphylococcal virulence factors. The patient became afebrile within the first 72 h of ICU admission. During the first 5 days of ICU stay, his PLT and WBC count reached its nadir. Finger desquamation mainly on his feet appeared on the 9th day of hospitalization, fulfilling at that stage five out of five clinical criteria for TSS [Fig. 2]. During the ICU stay, the patient presented acute renal failure and he received renal replacement therapy with hemofiltration for 21 days. The platelet count remained low and due to the increased risk of central nervous system (CNS) bleeding, the patient received additionally after PLTs transfusions, PLT growth factor, eltrombopag (75 mg 24 h), and dexamethasone (8 mg every 8 h) for 4 consecutive days, after having a hematologist expert consultation with the indication of persistent refractory thrombocytopenia. All drug doses were accordingly adjusted. The patient gradually improved over the following 2 months, and he was discharged from the hospital without sequelae.

**Table 1:** Laboratory data at admission

Parameter	Recorded value	Reference value
White blood cell count	0.80 × 10^3^/µL	4.50–7.50 × 10^3^/µL
Neutrophils	73%	42–74%
Lymphocytes	19%	18–50%
Hemoglobin	14.5 g/dL	11.3–15.2 g/dL
Hematocrit	46.1%	36–45%
Platelets	78 × 10^3^/µL	130–350 × 10^3^/µL
C-reactive protein	368 mg/L	≤ 10
Procalcitonin (PCT)	115,45 ng/ml	< 0.1 negative
Total protein	45 g/L	63–82 g/L
Albumin	26 g/L	35–50 g/L
Aspartate aminotransferase	20 U/L	38–126 U/L
Alanine aminotransferase	45 U/L	21–72 U/L
Lactate dehydrogenase	644 U/L	120–246 U/L
Creatine phosphokinase (CPK)	3405 U/L	55–170 U/L
Blood nitrogen urea	60 mg/dL	19–43 mg/dL
Creatinine	2.7 mg/dL	0.66–1.25 mg/dL
Sodium	140 mEq/L	137–145 mEq/L
Potassium	3.61 mEq/L	3.5–5.0 mEq/L
Glucose	136 mg/dL	74–106 mg/dL
D-Dimer	39,419 mg/dl	< 500 negative
Fibrinogen	838 mg/dl	180–350
APTT	55 s	23.9–34,9
Rapid influenza test	Positive for type B	

## Discussion and conclusion

The current patient was diagnosed with TSS, as he fulfilled all five diagnostic criteria proposed by the Council of State and Territorial Epidemiologists (USA) and Centers for Disease Control and Prevention (US) [12]. To the best of our knowledge this is the first adult case with a TSS syndrome induced by *Staphylococcus epider*mitis following an influenza type B infection, who had favorable progression and outcome.

According to the literature, both clinically and experimentally, bacterial complications (super-infections) are most pronounced after 5–7 days of acute influenza infection, which was evident also in our case [13]. Deciphering the mechanisms of bacterial superinfections (loss of the epithelial barrier function and altered innate immune defense), is of importance, to provide new diagnostic tools and therapeutic approaches. According to the literature influenza infection appears to prime the host airways for bacterial infection, whilst modifying and impairing immune responses in a number of ways. Viral induced immunosuppression can allow for a bacterial super infection, as host immune responses can be suppressed when immunologic cells are impaired during influenza infection and immune cell dysfunction can reduce the host’s ability to fight bacteria [14].

Typically, TSS is caused by *S. aureus* or *streptococcal* infections. Exotoxins secreted from *Staphylococcus aureus* or *Streptococcus pyogenes* might act as superantigens enhancing inflammation processes via cytokine storm release [5, 6]. However, the exact pathophysiological mechanism behind the presence of TSS induced by CoNS, remains largely unknown and warrants further investigation.

The true incidence of TSS by CoNS is an unexplored territory, and currently only few cases have been reported in the literature [Table 3]. Among them, a recently published case by Goda K et al. with TSS caused by a CoNS species (*Staphylococcus simulans*) after an episode of pneumococcal pneumonia associated with influenza [10]. Of interest, this case presented with high levels of inflammatory markers and cytokines (neopterin and IL-6), supporting the hypothesis of a cytokine storm release in CoNS TSS [10]. Similarly, Pomputius WF et al. reported a pediatric case of CoNS TSS due to *S. epidermitis* [11]. Although superantigen proteins were not isolated from the bloodstream of the child, a panel of four superantigen genes were finally detected in the plasma, suggesting that the CoNS found in urine, could be a causative agent inducing TSS.

**Table 2:** TTS criteria [12]

Clinical Criteria
Fever: temperature greater than or equal to 38.9 °C
Rash with diffuse macular erythroderma
Desquamation: 1–2 weeks after onset of rash
Hypotension: systolic blood pressure less than or equal to 90 mm Hg for adults or less than fifth percentile by age for children aged less than 16 years
Multisystem involvement (three or more of the following organ systems): o Gastrointestinal: vomiting or diarrhea at onset of illness o Muscular: severe myalgia or creatine phosphokinase level at least twice the upper limit of normal o Mucous membrane: vaginal, oropharyngeal, or conjunctival hyperemia o Renal: blood urea nitrogen or creatinine at least twice the upper limit of normal for laboratory or urinary sediment with pyuria (greater than or equal to 5 leukocytes per high-power field) in the absence of urinary tract infection o Hepatic: total bilirubin, alanine aminotransferase enzyme, or asparate aminotransferase enzyme levels at least twice the upper limit of normal for laboratory o Hematologic: platelets less than 100,000/mm^3^ o Central nervous system: disorientation or alterations in consciousness without focal neurologic signs when fever and hypotension are absent
Laboratory Criteria
Negative results on the following tests, if obtained: Blood or cerebrospinal fluid cultures (blood culture may be positive for Staphylococcus aureus) Negative serologies for Rocky Mountain spotted fever, leptospirosis, or measles
Case Classification
Probable A case which meets the laboratory criteria and in which four of the five clinical criteria described above are present
Confirmed A case which meets the laboratory criteria and in which all five of the clinical criteria described above are present, including desquamation, unless the patient dies before desquamation occurs

**Table 3:** Case series of published CoNS induced TSS in the recent literature

Author (year)	Age of patient/sex	CoN Strain isolated	TSS criteria	Treatment	Outcome	Comments
Goda K, et al. (2021) [10]	75 yrs/female	*Staphylococcus simulans*	4/5	Initial treatment meropenem (1 g every 8 h), vancomycin (1 g every 12 h, therapeutic drug monitoring: ≥15 µg/mL), and clindamycin (600 mg every 8 h). Targeted treatment: cefazolin (1 g q8h) and clindamycin (600 mg q8h), duration 14 days.	Not reported	Pneumococcal pneumonia and bacteremia from *S. simulans* following an influenza type A infection Increased levels of cytokines
Pomputius WF, et al. (2023 [11]	8yrs/ male	*Staphylococcus epidermidis*	5/5	vancomycin (hospital day 1–5), ceftriaxone (hospital day 1–8), clindamycin (hospital day 1–5), and acyclovir (hospital day 2–4). Doxycycline was begun on hospital day 4 and continued for a 10-day course, given concern forpossible *Chlamydia* or *Mycoplasma* encephalitis. No intravenous immunoglobulin or steroids were given.	survived	Positive urine culture of S. epidermidis. Four superantigen genes were detected in the plasma
Armeftis C, et al. (2023)	46yrs/ male	*Staphylococcus epidermidis*	5/5	meropenem (1 g every 8 h), vancomycin (1 g every 12 h), levofloxacin (750 mg 24 h), clindamycin (600 mg every 8 h), IVIG (2 g/kg) and hydrocortisone (100 mg every 8 h). The patient became afebrile within the first 72 h of ICU admission.	survived	TSS caused by Staphylococcus epidermitis following an influenza type B infection.

To conclude, we present a rare adult case TSS caused by coagulase-negative staphylococci (CoNS). It remains to be further evaluated how TSS is induced by the CoNS infection, with several mechanisms being proposed, including the presence of superantigens and hyper inflammation induced by cytokine mediators.

## Data Availability

Further patient’s data are available upon request to the Corresponding Author, Zoi Dorothea Pana, email: z.pana@euc.ac.cy, contact phone: +35,794,049,474.

## Abbreviations

CoNS: Coagulase-negative staphylococcus
CT: Computed tomography
MRSE: Methicillin-resistant Staphylococcus – epidermidis
S. simulans: Staphylococcus simulans
S. aureus: Staphylococcus aureus
PCR: Polymerase chain rection
TSS: Toxic shock syndrome
ICU: Intensive Care Unit
IVIG: Intravenous Immunoglobulin
WBC: White blood cell count
Plt: Platelet count
CRP: C reactive protein
CNS: Central nervous system
